# Association of Statin Use With Risk of Liver Disease, Hepatocellular Carcinoma, and Liver-Related Mortality

**DOI:** 10.1001/jamanetworkopen.2023.20222

**Published:** 2023-06-26

**Authors:** Mara Sophie Vell, Rohit Loomba, Arunkumar Krishnan, Kirk J. Wangensteen, Jonel Trebicka, Kate Townsend Creasy, Christian Trautwein, Eleonora Scorletti, Katharina Sophie Seeling, Leonida Hehl, Miriam Daphne Rendel, Inuk Zandvakili, Tang Li, Jinbo Chen, Marijana Vujkovic, Saleh Alqahtani, Daniel James Rader, Kai Markus Schneider, Carolin Victoria Schneider

**Affiliations:** 1 Gastroenterology, Metabolic Diseases, and Intensive Care, Department of Medicine III, University Hospital RWTH Aachen, Aachen, Germany; 2 Division of Gastroenterology, University of California, San Diego, La Jolla; 3 Section of Gastroenterology and Hepatology, West Virginia University School of Medicine, Morgantown; 4 Department of Genetics, Perelman School of Medicine, University of Pennsylvania, Philadelphia; 5 Division of Gastroenterology and Hepatology, Mayo Clinic, Rochester, Minnesota; 6 Medical Clinic B, Gastroenterology, Hepatology, Endocrinology, Clinical Infectiology, University Hospital Münster, Münster, Germany; 7 Department of Biobehavioral Health Sciences, School of Nursing, University of Pennsylvania, Philadelphia; 8 Department of Biostatistics, Epidemiology and Informatics, Perelman School of Medicine, University of Pennsylvania, Philadelphia; 9 Division of Gastroenterology and Hepatology, Johns Hopkins University School of Medicine, Baltimore, Maryland; 10 Liver Transplant Center, King Faisal Specialist Hospital & Research Center, Riyadh, Saudi Arabia; 11 Institute for Translational Medicine and Therapeutics, Perelman School of Medicine, University of Pennsylvania, Philadelphia

## Abstract

**Importance:**

Given the burden of chronic liver disease on the health care system, more information on the hepatoprotective association of statins in the general population is needed.

**Objective:**

To examine whether regular statin use is associated with a reduction in liver disease, particularly hepatocellular carcinoma (HCC) and liver-related deaths, in the general population.

**Design, Setting, and Participants:**

This cohort study used data from the UK Biobank (UKB) (individuals aged 37-73 years) collected from baseline (2006-2010) to the end of follow-up in May 2021, from the TriNetX cohort (individuals aged 18-90 years) enrolled from baseline (2011-2020) until end of follow-up in September 2022, and from the Penn Medicine Biobank (PMBB) (individuals aged 18-102 years) with ongoing enrollment starting in 2013 to the end of follow-up in December 2020. Individuals were matched using propensity score matching according to the following criteria: age, sex, body mass index, ethnicity, diabetes with or without insulin or biguanide use, hypertension, ischemic heart disease, dyslipidemia, aspirin use, and number of medications taken (UKB only). Data analysis was performed from April 2021 to April 2023.

**Exposure:**

Regular statin use.

**Main Outcomes and Measures:**

Primary outcomes were liver disease and HCC development as well as liver-associated death.

**Results:**

A total of 1 785 491 individuals were evaluated after matching (aged 55 to 61 years on average, up to 56% men, and up to 49% women). A total of 581 cases of liver-associated death, 472 cases of incident HCC, and 98 497 new liver diseases were registered during the follow-up period. Individuals were aged 55-61 years on average, with a slightly higher proportion of men (up to 56%). In UKB individuals (n = 205 057) without previously diagnosed liver disease, statin users (n = 56 109) had a 15% lower hazard ratio (HR) for the association of developing a new liver disease (HR, 0.85; 95% CI, 0.78-0.92; *P* < .001). In addition, statin users demonstrated a 28% lower HR for the association with liver-related death (HR, 0.72; 95% CI, 0.59-0.88; *P* = .001) and a 42% lower HR for the development of HCC (HR, 0.58; 95% CI, 0.35-0.96; *P* = .04). In TriNetX individuals (n = 1 568 794), the HR for the association of HCC was reduced even further for statin users (HR, 0.26; 95% CI, 0.22-0.31; *P* = .003). The hepatoprotective association of statins was time and dose dependent, with a significant association in PMBB individuals (n = 11 640) for incident liver diseases after 1 year of statin use (HR, 0.76; 95% CI, 0.59-0.98; *P* = .03). Taking statins was particularly beneficial in men, individuals with diabetes, and individuals with a high Fibrosis-4 index at baseline. Carriers of the heterozygous minor allele of *PNPLA3* rs738409 benefited from statin use and had a 69% lower HR for the association with HCC (UKB HR, 0.31; 95% CI, 0.11-0.85; *P* = .02).

**Conclusions and Relevance:**

This cohort study indicates substantial preventive associations of statins against liver disease, with an association with duration and dose of intake.

## Key Points

**Question:**

Is regular statin intake associated with a reduced rate of new liver diseases and liver-related death in the general population?

**Findings:**

This cohort study, involving more than 1.7 million people after matching, found that regular use of statins was associated with a 15% lower hazard ratio of new-onset liver disease and a 28% lower hazard ratio for liver-related deaths compared with no statin use. Moreover, a decrease in hazard ratio of up to 74% was demonstrated for hepatocellular carcinoma in regular statin users compared with non–statin users.

**Meaning:**

These findings suggest that statins may be a therapeutic option for liver disease prevention and should be examined in further studies.

## Teaser

This cohort study investigates the association of regular statin use with liver-associated morbidity and liver-related mortality in the general population.

## Introduction

Worldwide, more than 2 million people die of liver-related deaths every year.^1,2^ In view of the increasing health crisis and limited options for liver disease prevention, drug repurposing represents an appealing prospect. Statins, as 3-hydroxy-3-methylglutaryl coenzyme A (HMG-CoA) reductase inhibitors, possess HMG-CoA–dependent and HMG-CoA–independent mechanisms, including antiproliferative,^3,4^ antimetastatic,^5^ proapoptotic,^6,7^ antiangiogenic,^7,8^ and immunomodulatory^9^ modes of action. A previous study^10^ found that statins reduce the risk of hepatocellular carcinoma (HCC) occurrence through pleiotropic effects. Furthermore, statins have been found to reduce hepatic inflammation by inhibiting the prenylation of small guanosine triphosphate hydrolases to mitigate oxidative stress.^11,12^ In addition, the low-density lipoprotein–lowering effect of statins may also exhibit hepatoprotective effects with regard to the development of steatosis, although further experimental studies are required.^13,14^

Furthermore, the administration of statins can be effective in portal hypertension.^15^ By inhibiting RhoA translocation, statins reduce RhoA kinase activity, which counteracts further vasoconstriction.^14,16^ Moreover, statins improve endothelial dysfunction by enhancing endothelial nitric oxide synthesis activity and nitric oxide availability.^14,17^ However, information is lacking on the relevance of these effects in the general population, especially in individuals without a history of liver-related diseases. In this study, we investigated the association of statin use with both liver-associated morbidity and liver-related mortality in more than 1.7 million individuals from 3 cohorts.

## Methods

This cohort study used data collected from the UK Biobank (UKB), the Penn Medicine Biobank (PMBB), and the TriNetX research network. Protocols for the PMBB study were approved by the institutional review board at Penn Medicine. Written informed consent was obtained. The UKB study has approval from the North West Multi-centre Research Ethics Committee. TriNetX received a waiver from the Western Institutional Review Board as a federated network because it only includes aggregated counts and deidentified information statistical summaries. This study conformed to the Strengthening the Reporting of Observational Studies in Epidemiology (STROBE) reporting guideline.

### UK Biobank

The UKB was authorized by the North West Multi-centre Research Ethics Committee and involved 502 511 volunteers aged 37 to 73 years starting in 2006. The end of follow-up was defined as death or end of data collection in May 2021. The mean (SD) follow-up time was 11.9 (1.9) years. Diagnoses were coded in accordance with the *International Statistical Classification of Diseases and Related Health Problems, Tenth Revision* (*ICD-10*). The hospital admission code was used to register *ICD-10* codes and transmit them to the UKB. Using the National Death Registry, we collected data on the patient’s age and primary *ICD-10* diagnosis that led to death.

#### Medication

Medication was collected at the time of entry into the study. The type and number of prescription drugs were listed by the participant. Medications taken regularly as part of a daily, weekly, or monthly routine should be listed by the participant, so regular use can be assumed for statin users. Medications in the UKB have been validated and analyzed before.^18^ We included single preparations of statins in the analyses and report the used UKB characteristics in eTable 1 in Supplement 1.

#### Exclusion Criteria

Criteria for exclusion from the UKB cohort were missing body mass index (BMI), age, and survival data; HIV (*ICD-10* codes B20-B24); or chronic hepatitis (*ICD-10* code B18) (Figure). We excluded individuals with any liver disease diagnosis (*ICD-10* codes K70, alcohol-associated liver disease; K71, toxic liver disease; K72, hepatic failure, not elsewhere classified; K73, chronic hepatitis, not elsewhere classified; K74, fibrosis and cirrhosis of liver; K75, other inflammatory liver diseases; K76, other diseases of liver; and K77, liver disorders in diseases classified elsewhere) or HCC (*ICD-10* code C22.0) at baseline as well as pathological alcohol consumption (>60 g/d for men and >40 g/d for women).^19^ Alcohol consumption and quantity were evaluated using a cascading questionnaire. First, it was determined how often alcoholic beverages were consumed. The second question was asked when a choice other than never was given and referred to the amount of alcohol consumed in a day. A drink was evaluated by the UKB as 1 U of alcohol. The third question asked how many alcohol-containing drinks were consumed on a typical day when drinking. Response options included 1 or 2; 3 or 4; 5 or 6; 7, 8, or 9; or 10 or more.^20^

**Figure.**
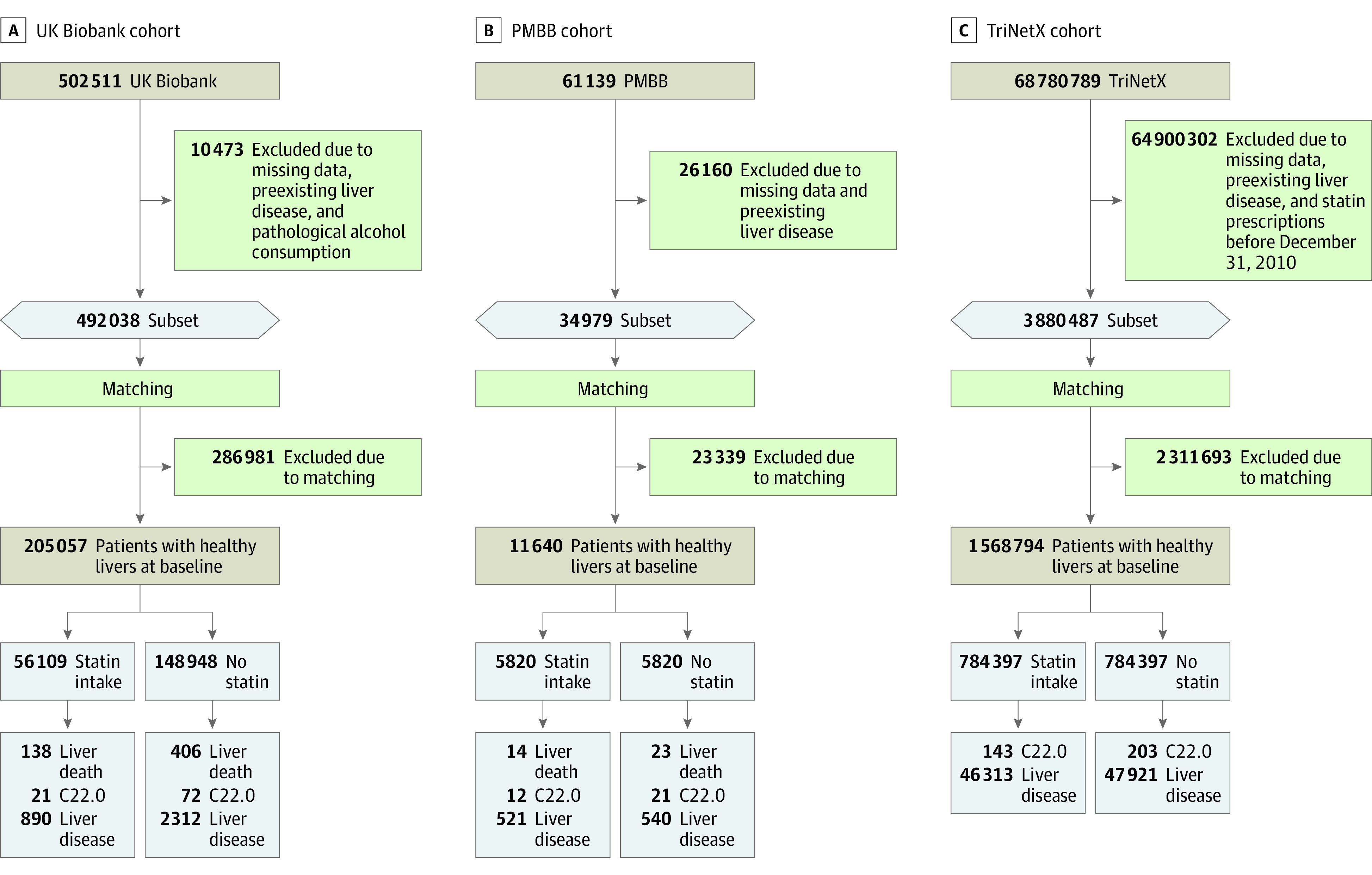
Flowchart of the UK Biobank, Penn Medicine Biobank (PMBB), and TriNetX Cohorts C22.0 indicates hepatocellular carcinoma.

### Penn Medicine Biobank

The University of Pennsylvania Health System provided the PMBB data set, which was approved by an institutional review board. Individuals gave written informed consent to access their electronic health records (EHRs) starting in 2013, with ongoing enrollment. Using medical record data, we identified diagnoses based on *ICD-10* codes. The latter included the same diagnoses used in the UKB. Additional patient characteristics were obtained from the EHR. The linkage to the EHR enabled the PMBB to receive death data and *ICD-10* diagnoses leading to death. The study included 61 139 people aged 18 to 102 years, of whom 5820 were taking statins regularly (Figure). The end of follow-up was death or the end of data collection in December 2020. Mean (SD) follow-up time was 4.7 (2.3) years.

#### Medication

We calculated the duration of intake for prescribed medications from the EHR information on the prescription date, amount to dispense, and number of refills. In the absence of other information, we regarded a dispense amount of 30 as the minimum duration. We did not include any patients who had a shorter duration of intake in the analysis and only included patients with the requested or a longer duration of intake in the statin group.

#### Exclusion Criteria

Criteria for exclusion for the PMBB were incorrect or missing BMI, age, and survival data; HIV infection (*ICD-10* codes B20-B24); and chronic hepatitis (*ICD-10* code B18) (Figure). To define a cohort of patients without previously diagnosed liver disease as in the UKB, we used either the time of enrollment or, in the case of statin use, the time of initial use as the reference time. We excluded an additional 230 patients with preexisting liver disease (*ICD-10* codes K70-K77). Another 6 participants were excluded because of a preexisting HCC diagnosis (Figure). Data on alcohol intake were not available.

### TriNetX

The TriNetX research network is a federated multicenter research network that provides real-time access to an anonymized data set from participating health care organizations’ EHRs. The Western Institutional Review Board has granted a waiver to TriNetX as a federated network. We identified all individuals aged 18 to 90 years who were newly prescribed treatment with statins and nonusers between January 2011 and December 2020. Follow-up ended in September 2022. The cohort included 784 397 individuals per group after matching. Clinical variables and death data are directly retrieved from the EHR via an integrated system of clinical records. Individuals had no diagnosis of liver disease (*ICD-10* codes K70-K77) or HCC (*ICD-10* code C22.0) at baseline.

#### Medication

Exposure to statins was lagged by 6 months to account for an adequate duration of drug use and possible latency period and to minimize reverse causality.^21^ First contact with a statin was defined as an index event. Prescriptions of statins before December 31, 2010, were excluded, so only new users were included.

#### Exclusion Criteria

Criteria for exclusion from the TriNetX cohort were missing BMI, age, and survival data; HIV infection; liver disease before December 2010; and the use of statins before the index date. Data on alcohol intake were not available. Further methods can be found in the eAppendix in Supplement 1.

### Primary and Secondary Outcomes

Primary outcomes of the study were incident liver disease, HCC, and liver-related mortality. Individuals had no preexisting liver disease (*ICD-10* codes K70-K77 or C22.0) at baseline examination. We considered any new diagnosis of *ICD-10* codes K70 to K77 after baseline as incident liver disease. Hepatocellular carcinoma was defined as C22.0. Liver-related death was defined as death by *ICD-10* codes K70 to K77 or C22.0. Secondary outcomes were drug-induced myopathy (*ICD-10* code G72.0) and diabetes type 2 (*ICD-10* code E11).

### Sensitivity Analyses

#### Metabolic Profile

In the UKB, nuclear magnetic resonance–based metabolomic profiling was performed in a subgroup of 105 348 participants. Of the 168 metabolites provided by the UKB, we focused on 64 metabolites of the lipid profile, which could be assigned to the following groups: lipoprotein subclasses particle concentrations, cholesterol, triglycerides, fatty acids, phospholipids, and apolipoproteins. We also considered the glucose levels in more detail. We compared the metabolic profiles of statin users at baseline with nonusers after matching (eFigure 2 in Supplement 1). To compare the metabolites in the treatment and nontreatment groups, we calculated the log fold change. The Bonferroni correction was performed to avoid the type I error caused by multiple testing. A value of -log(p/64)>3.1 was considered statistically significant.

#### Intake Duration in PMBB

We performed sensitivity analyses to account for differences in intake duration. We excluded an intake duration of less than 30 days to assume sufficient intake period. We selected periods of 30 days or more, 90 days or more, 180 days or more, and 360 days or more as the respective minimum intake durations.

#### IPTW Model

We performed an additional analysis using inverse probability of treatment weighting (IPTW). We computed the IPTW weights based on the propensity scores and treatment assignment and used a survey design to account for weighting and clustering in the data. We then used Cox proportional hazards regression models to estimate the effect of statin use on each outcome of interest, including liver death, using the whole cohort without exclusions because of matching. The IPTW was used to minimize bias because of confounding when assessing the association of a treatment with the different outcomes.

#### Genetic Disposition

We examined the association of statin intake with liver disease outcomes in carriers of known liver disease–associated gene variants in the UKB. Genotyping was conducted in 488 377 participants using the UKB Axiom Array. Haplotype Reference Consortium and UK10K were used as a reference sample.^18,20^ We considered the following genes: rs738409 *PNPLA3* (OMIM 609567), rs58542926 *TM6SF2* (OMIM 606563), rs72613567 *HSD17B13* (OMIM 612127), rs2642438 *MTARC1* (OMIM 614126), and rs28929474 SE*RPINA1* (OMIM 107400). Because statins are predominantly metabolized via the isoenzyme *CYP3A4* (OMIM 124010), we examined the association of the variant CYP3A4*22 (rs35599367), which results in partial loss of function,^22^ on liver disease outcomes in the UKB in a 2:1 matching for carriers (heterozygous and homozygous) of this variant to controls.^23,24^

### Statistical Analysis

Data analysis was performed from April 2021 to April 2023. A Cox proportional hazards regression model was used to evaluate the association between statin use and outcomes adjusted for age, sex, BMI, ethnicity (self-reported ethnicity was included because it could affect the frequency of statin prescriptions), and number of medications (UKB only). For specific liver diseases, we performed a competing risk analysis integrating all liver diseases except the considered one as 1 competing event. The Fine and Gray regression model with non–liver-related death as a competing event adjusted for age, sex, BMI, ethnicity, and number of medications (UKB only) was used to assess the association between statin use and liver-related death.

We verified that all variables were normally distributed. The propensity score matching approach was used to balance the characteristics of both groups at baseline (treatment group and nontreatment group) in all cohorts. The propensity scores considered sociodemographic data, health status, and medications known to affect the likelihood of statin use. Among them were age, sex, BMI, ethnicity, diabetes (*ICD-10* code E11) with or without insulin (UKB only) or biguanide intake, hypertension (*ICD-10* code I10), ischemic heart disease (*ICD-10* codes I20 and I25), dyslipidemia (*ICD-10* code E78), and aspirin intake (and the number of medications taken, which was only available in the UKB). The UKB analyses were performed with a ratio of 5 in R software, version 4.1.2 (R Foundation for Statistical Computing) (at least 1 control patient and a maximum of 5 control individuals were matched to a patient taking a statin). We also established a 1:1 matching in the PMBB and TriNetX cohorts. Additional information can be found in the eMethods in Supplement 1.

Data were expressed as mean (SD). Hazard ratios (HRs) are given with their 95% CIs. A 2-sided *P* < .05 was considered significant. E-values were calculated using R software, version 4.1.2. Statistical analyses were performed using R, version 4.1.2; SPSS, version 27 (IBM Corp); and GraphPad Prism, version 8.0.1 (GraphPad Software Inc).

## Results

A total of 1 785 491 individuals were evaluated after matching. Individuals were aged 55 to 61 years on average, with a slightly higher proportion of men (up to 56% vs up to 49% women), 70% to 95% were White, whereas 2% to 3% were Asian, and 1% to 22% were Black. A total of 581 cases of liver-associated death, 472 cases of incident HCC, and 98 497 new liver diseases were registered during the follow-up period.

### Association of Statin Intake With Protection From Liver Disease in the UKB

In the UKB individuals (n = 205 057) without previously diagnosed liver disease, after matching, we identified 56 109 individuals regularly receiving statins and 148 948 controls (Figure). Table 1 gives their baseline characteristics. Individuals in the UKB had a mean (SD) age of 61 (6.3) years; 44% were female and 56% were male (Table 1). Nonusers in the UKB had a mean (SD) age of 60 (6.6) years; 47% were female and 53% were male (Table 1). The proportion of individuals with diabetes was higher among statin users (14%) than nonusers (6%) (Table 1).

**Table 1.** Baseline Characteristics of the Matched Cohort^a^

Characteristic	No statin intake	Statin intake	Standardized mean difference before PS	Standardized mean difference after PS
**Patients without prior liver disease in the UKB**				
No.	148 948	56 109	NA	NA
Age, mean (SD), y	60 (6.6)	61 (6.3)	0.8	0.1
Sex				
Male	78 774 (53)	31 542 (56)	0.4	0.0
Female	70 174 (47)	24 567 (44)	0.4	0.0
BMI, mean (SD)	28.2 (5.0)	28.9 (4.8)	0.5	0.0
Ethnicity				
Asian	2604 (2)	1606 (3)	0.0	0.0
Black	2209 (2)	714 (1)	0.0	0.0
White	141 800 (95)	52 753 (94)	0.0	0.0
Other^b^	2335 (2)	1036 (2)	0.0	0.0
No. of medications, mean (SD)	4 (3.0)	5 (3.0)	1.3	0.0
Diabetes type 2 (*ICD-10* code E11)	8941 (6)	7590 (14)	0.6	0.0
Arterial hypertension (*ICD-10* code I10)	46 838 (31)	24 777 (44)	0.9	0.0
Disorders of lipoprotein metabolism and other lipidemias (*ICD-10* code E78)	20 318 (14)	15 211 (27)	0.9	0.0
**Patients without prior liver disease in the PMBB**				
No.	5820	5820	NA	NA
Age, mean (SD), y	60 (14)	60 (13)	1.1	0.1
Sex				
Male	3140 (54)	3200 (55)	0.5	0.0
Female	2680 (46)	2620 (45)	0.5	0.0
BMI, mean (SD)	29.6 (8.3)	29.6 (6.7)	0.3	0.0
Ethnicity				
American Indian or Native American	1 (<1)	6 (<1)	0.0	0.0
Asian	93 (2)	102 (2)	0.0	0.0
Black	1270 (22)	1288 (22)	0.0	0.0
White	4124 (71)	4081 (70)	0.0	0.0
Other^b^	332 (6)	343 (6)	0.0	0.0
Diabetes type 2 (*ICD-10* code E11)	1141 (20)	1559 (27)	0.8	0.1
Arterial hypertension (*ICD-10* code I10)	3384 (58)	3577 (62)	1.0	0.0
Disorders of lipoprotein metabolism and other lipidemias (*ICD-10* code E78)	3538 (61)	3838 (66)	1.8	0.1
**Patients without prior liver disease in TriNetX**				
No.	784 397	784 397	NA	NA
Age, mean (SD), y	56 (11)	55 (11)	0.4	0.1
Sex				
Male	399 402 (51)	424 927(54)	0.3	0.1
Female	384 995 (49)	359 470 (46)	0.3	0.1
BMI, mean (SD)	29.9 (6.9)	31.1 (6.7)	0.2	0.1
Diabetes type 2 (*ICD-10* code E11)	57 118 (7)	69 689 (9)	0.0	0.1
Arterial hypertension (*ICD-10* code I10)	182 107 (23)	202 709 (26)	0.1	0.1
Disorders of lipoprotein metabolism and other lipidemias (*ICD-10* code E78)	104 561 (13)	124 461 (16)	0.6	0.1

Abbreviations: BMI, body mass index (calculated as weight in kilograms divided by height in meters squared); *ICD-10*, *International Statistical Classification of Diseases and Related Health Problems, Tenth Revision*; NA, not applicable; PMBB, Penn Medicine Biobank; PS, propensity scoring; UKB, UK Biobank.

^a^ Data are presented as percentages unless otherwise indicated.

^b^ Other ethnicities include Chinese, mixed ethnicity, or other ethnic group in the UKB and Hispanic and Pacific Islander, other, or unknown in the PMBB.

A total of 138 liver-related deaths, 21 new diagnoses of HCCs, and 890 new diagnoses of liver diseases were reported among statin users, whereas 406 liver-related deaths, 72 HCCs, and 2312 liver diseases were noted for controls (Figure). Statin users had a 15% (HR, 0.85; 95% CI, 0.78-0.92; *P* < .001) lower HR of developing incident liver disease compared with nonusers (Table 2; eFigure 1 in Supplement 1). Additional analyses of the different liver disease subdiagnoses showed a significantly lower HR for *ICD-10* codes of alcohol-associated liver disease (*ICD-10* code K70) and other diseases of the liver (*ICD-10* code K76), which includes nonalcoholic fatty liver disease (NAFLD). Interestingly, there was a 30% lower HR for the development of incident cirrhosis among statin users (HR, 0.70; 95% CI, 0.56-0.86; *P* < .001) (Table 2). Similarly, statin users had a 42% decreased HR (HR, 0.58; 95% CI, 0.35-0.96; *P* = .04) for incident HCC and were 63% less likely to undergo liver transplantation compared with nonusers (HR, 0.37; 95% CI, 0.14-0.96; *P* = .04) (Table 2). Inclusion of additional covariates (daily alcohol consumption, diet, and socioeconomic status) had only a negligible effect on the outcomes: only alcohol-associated liver disease did not remain significant after this correction (eTables 2 and 3 in Supplement 1). To further corroborate the results and validate our statistical approach, we developed an IPTW model to adjust for unmeasured confounding and selection bias. The IPTW method with the Cox proportional hazards regression model for competing risks showed a decrease in the HR for the development of new liver disease in statin users of 31% (HR, 0.69; 95% CI, 0.48-0.97; *P* = .03) (eTable 4 in Supplement 1). Finally, we examined liver-associated death in statin users and found a 28% lower hazard ratio (HR, 0.72; 95% CI, 0.59-0.88; *P* = .001) compared with nonusers (Table 2; eFigure 1 in Supplement 1).

**Table 2.** Statin Use and the Development of Liver Disease, Hepatocellular Carcinoma, and Liver-Related Mortality in the UK Biobank

Event and treatment group	No. with event/total No.	Hazard ratio (95% CI)	*P* value	E-value
New liver disease (*ICD-10* codes K70-K77)				
No statin intake	2312/148 948	1 [Reference]	NA	NA
Statin intake	890/56 109	0.85 (0.78-0.92)	<.001^a^	1.63
Subdiagnoses^b^				
Alcohol-associated liver disease (*ICD-10* code K70)	95/56 109	0.74 (0.58-0.94)	.02^a^	2.06
Toxic liver disease (*ICD-10* code K71)	5/56 109	0.42 (0.17-1.05)	.06	NA
Hepatic failure, not elsewhere classified (*ICD-10* code K72)	71/56 109	0.89 (0.66-1.18)	.40	NA
Chronic hepatitis, not elsewhere classified (*ICD-10* code K73)	11/56 109	1.12(0.55-2.28)	.80	NA
Fibrosis and cirrhosis of liver (*ICD-10* code K74)	116/56 109	0.70 (0.56-0.86)	<.001^a^	2.23
Other inflammatory liver diseases (*ICD-10* code K75)	111/56 109	0.88 (0.71-1.10)	.30	NA
Other diseases of the liver (*ICD-10* code K76)	683/56 109	0.86 (0.79-0.94)	.001^a^	1.59
Liver disorders in diseases classified elsewhere (*ICD-10* code K77)	1/56 109	0.56 (0.05-6.84)	.70	NA
Liver cell carcinoma (*ICD-10* code C22.0)	21/56 109	0.58 (0.35-0.96)	.04^a^	2.84
Liver transplant status (*ICD-10* code Z94.4)	5/56 109	0.37 (0.14-0.96)	.04^a^	4.89
Liver-related death				
No statin intake	406/148 948	1.00 [Reference]	NA	NA
Statin intake	138/56 109	0.72 (0.59-0.88)	.001^a^	2.12
Drug-induced myopathy (*ICD-10* code G72.0)				
No statin intake	17/148 948	1 [Reference]	NA	NA
Statin intake	5/56 109	0.62 (0.23-1.65)	.30	NA
Diabetes (*ICD-10* code E11)^c^				
No statin intake	8615/148 948	1 [Reference]	NA	NA
Statin intake	7871/56 109	2.04 (1.97-2.10)	<.001^a^	3.50

Abbreviations: *ICD-10*, *International Statistical Classification of Diseases and Related Health Problems, Tenth Revision*; NA, not applicable.

^a^ Significant *P* value.

^b^ For subdiagnoses, only individuals taking statins are listed, with hazard ratios and *P* values calculated consistently compared with individuals not taking statins.

^c^ Adjustment was made to matching for the UK Biobank described in the Methods by excluding E11 from the propensity score matching for this analysis.

### Initiation of New Statin Treatment and the Development of Incident Liver Disease in TriNetX

After establishing a clear association between statin use and liver disease protection in the UKB, we turned to the TriNetX cohort to study whether this association also holds in individuals starting a new medication with statins. The TriNetX cohort included 784 397 new users of statins and 784 397 nonusers after matching. The mean (SD) age of individuals in the TriNetX cohort was 56 (11) years, 46% were female, and 54% were male (Table 1). The mean (SD) age of nonusers in the TriNetX cohort was 56 (11) years; 49% were female, and 51% were male (Table 1). Statin users and nonusers had a more balanced distribution of medical conditions. Diabetes was diagnosed in 9% of statin users and 7% of nonusers (Table 1). In total, 94 234 incident liver diseases were reported, of which 47 921 occurred among nonusers and 46 313 among new users (Figure). New users of statins showed a 4% lower HR for the association for new liver diseases (HR, 0.96; 95% CI, 0.95-0.97; *P* < .001) (Table 3). Regarding individual liver diseases, there was a significant association for alcohol-related liver disease (HR, 0.56; 95% CI, 0.53-0.59; *P* < .001) and for toxic liver disease (HR, 0.69; 95% CI, 0.61-0.78; *P* < .001), hepatic failure (HR, 0.63; 95% CI, 0.60-0.66; *P* < .001), chronic hepatitis (HR, 0.84; 95% CI, 0.74-0.96; *P* = .003), and cirrhosis (HR, 0.70; 95% CI, 0.68-0.72; *P* < .001) in statin users (Table 3). The decrease in HR for developing new-onset HCC was 74% in statin users (HR, 0.26; 95% CI, 0.22-0.31; *P* = .003) (Table 3).

**Table 3.** Statin Intake and the Development of Incident Liver Disease, Hepatocellular Carcinoma, and Liver-Related Mortality in Individuals Without Prior Liver Disease in the TriNetX Cohort^a^

Event and treatment group	No. with event/total No.	Hazard ratio (95% CI)	*P* value	E-value
Liver disease (*ICD-10* codes K70-K77)				
No statin intake	47 921/784 397	1 [Reference]	NA	NA
Statin intake	46 313/784 397	0.96 (0.95-0.97)	<.001^b^	1.25
Subdiagnoses^c^				
Alcohol-associated liver disease (*ICD-10* code K70)	2573/784 397	0.56 (0.53-0.59)	<.001^b^	2.97
Toxic liver disease (*ICD-10* code K71)	439/784 397	0.69 (0.61-0.78)	<.001^b^	2.26
Hepatic failure, not elsewhere classified (*ICD-10* code K72)	2743/784 397	0.63 (0.60-0.66)	<.001^b^	2.55
Chronic hepatitis, not elsewhere classified (*ICD-10* code K73)	432/784 397	0.84 (0.74-0.96)	.003^b^	1.67
Fibrosis and cirrhosis of liver (*ICD-10* code K74)	7540/784 397	0.70 (0.68-0.72)	<.001^b^	2.21
Other inflammatory liver diseases (*ICD-10* code K75)	8360/784 397	0.95 (0.92-0.98)	<.001^b^	1.29
Other diseases of liver (*ICD-10* code K76)	38 590/784 397	0.98 (0.97-1.00)	<.001^b^	1.16
Liver disorders in diseases classified elsewhere (*ICD-10* code K77)	46/784 397	0.72 (0.49-1.06)	.07	NA
Liver cell carcinoma (*ICD-10* code C22.0)	143/784 397	0.26 (0.22-0.31)	.003^b^	7.15

Abbreviations: *ICD-10*, *International Statistical Classification of Diseases and Related Health Problems, Tenth Revision*; NA, not applicable.

^a^ Matching was performed at a ratio of 1:1.

^b^ Significant *P* value.

^c^ For subdiagnoses, only individuals taking statins are listed, with hazard ratios and *P* values calculated consistently compared with individuals not taking statins.

### Association Between Liver Disease Risk and Duration of Statin Intake in the PMBB

To further evaluate the association with intake duration, we performed sensitivity analyses to account for differences in statin intake duration in the comparably small but ethnically diverse PMBB. We selected a cohort of 5820 statin users and 5820 nonusers. The mean (SD) age was 60 (13) years, 45% were women, and 55% were men (Table 1). The mean (SD) age of nonusers in the PMBB was 60 (14) years; 46% were women, and 54% were men (Table 1). Diabetes was diagnosed in 27% of statin users and in 20% of nonusers. In the statin user group, a total of 14 liver-related deaths, 12 HCCs, and 521 new liver-related diagnoses were recorded. Among nonusers, 23 liver-associated deaths, 21 HCCs, and 540 new liver diagnoses occurred (Figure). The association between statin use and liver disease protection depended on the intake duration. A short statin intake period (30-180 days) showed no significant association, but after 360 days of statin treatment, the HR for new liver diseases was reduced by 24% (HR, 0.76; 95% CI, 0.59-0.98; *P* = .03) (Table 4).

**Table 4.** Time-Dependent Intake of Statins and Risk of Incident Liver Disease, Hepatocellular Carcinoma, and Liver-Related Mortality in Individuals Without Prior Liver Disease in the Penn Medicine Biobank^a^

Event and treatment group	No. with event/total No.	Hazard ratio (95% CI)	*P* value	E-value
New liver disease (*ICD-10* codes K70-K77)				
No statin intake	540/5820	1 [Reference]	NA	NA
Statin intake	521/5820	0.94 (0.83-1.06)	.30	NA
Time-dependent analyses				
Statin intake ≥30 d	516/5771	0.94 (0.83-1.06)	.30	NA
Statin intake ≥90 d	194/1930	0.94 (0.80-1.11)	.50	NA
Statin intake ≥180 d	104/1105	0.82 (0.67-1.01)	.07	NA
Statin intake ≥360 d	68/750	0.76 (0.59-0.98)	.03^b^	1.95
Liver-related death^c^				
No statin intake	23/5820	1 [Reference]	NA	NA
Statin intake	14/5820	0.60 (0.31-1.17)	.10	NA
Event hepatocellular carcinoma^c^				
No statin intake	21/5820	1 [Reference]	NA	NA
Statin intake	12/5820	0.56 (0.27-1.15)	.10	NA

Abbreviations: *ICD-10*, *International Statistical Classification of Diseases and Related Health Problems, Tenth Revision*; NA, not applicable.

^a^ Matching was performed at a ratio of 1:1.

^b^ Significant *P* value.

^c^ Not enough end points to perform time-dependent analyses.

### Statin Bioavailability and Liver Disease in the UKB

We investigated whether the CYP3A4*22 (rs35599367) variant, which reduces the metabolism of statins, affects liver disease outcomes in people who take statins (eTable 5 in Supplement 1). We found an even stronger association in incident liver diseases among homozygous/heterozygous minor allele carriers (HR, 0.68; 95% CI, 0.51-0.89; *P* = .005) (eTable 5 in Supplement 1). Together, these data suggest a dose-dependent association.

### Association Between Individual Genetic Risk for Liver Disease and Statin Use in the UKB

We next investigated whether genetic modifiers of liver disease affect the outcomes linked to statin intake. Single-nucleotide variants (SNVs) in *PNPLA3* and *TM6SF2* have been linked to NAFLD, cirrhosis, and HCC,^25,26,27^ whereas SNVs in *HSD17B13* and *MTARC1* reduce fat accumulation and protect against liver disease.^28,29,30^ In previous studies,^31,32,33^ SE*RPINA1* rs28929474 showed a significant association with liver disease severity.

The association for liver disease protection among statin users was evident for HCC among carriers heterozygous for the *PNPLA3* rs738409 risk allele (HR, 0.31; 95% CI, 0.11-0.85; *P* = .02) (eTable 6 in Supplement 1). For liver protective variants, we found a more pronounced association of statins with liver disease protection in noncarriers of the protective variants (eTable 6 in Supplement 1).

### Common Risk Factors for the Development of Liver Disease in the UKB

After studying genetic risk factors, we turned to common risk factors for liver disease development. Sex-specific analyses showed that men may derive greater benefit from statin intake, with a significant association for all liver disease end points, especially for HCC (HR, 0.53; 95% CI, 0.30-0.95; *P* = .03) (eTable 6 in Supplement 1). We found no statin benefits for women. Among subgroups based on the Fibrosis-4 (FIB-4) index,^34^ an ascending decrease in the HR for developing new liver disease was observed with an increasing index. In individuals with a FIB-4 index greater than 2.67, statin users showed a 30% lower HR (HR, 0.70; 95% CI, 0.55-0.90; *P* = .006) (eTable 6 in Supplement 1). Finally, in individuals with diabetes, we found a significant association between statin use and new liver disease (HR, 0.66; 95% CI, 0.57-0.76; *P* < .001) and liver-related death (HR, 0.61; 95% CI, 0.42-0.89; *P* = .01) (eTable 6 in Supplement 1).

### Metabolic Profile of Statin Users Compared With Nonusers in the UKB

The metabolic profiles of statin users vs nonusers are shown in eFigure 2 in Supplement 1. We observed a significant association for very low-density lipoprotein (no statin, 0.9 mmol/L; statin, 0.7 mmol/L; −19.56%; *P* < .001), low-density lipoprotein (no statin, 2.0 mmol/L; statin, 1.5 mmol/L; −21.54%; *P* < .001), and very large high-density lipoprotein (no statin, 0.00022 mmol/L; statin, 0.00018 mmol/L; −19.89%; *P* < .001) in statin users (eTable 7 in Supplement 1). We further found a significant association for total cholesterol in statin users (no statin, 5.2 mmol/L; statin, 3.8 mmol/L; −18.38%; *P* < .001).

### Statin-Associated Risks in the UKB

Statin users did not have an increased HR of developing drug-associated myopathy (HR, 0.62; 95% CI, 0.23-1.65; *P* = .30) (Table 2). Regarding the development of diabetes, statin users showed a significant increase (HR, 2.04; 95% CI, 1.97-2.10; *P* < .001) (Table 2) and an increased glucose level in the metabolomic analysis (eTable 7 in Supplement 1).

## Discussion

In this cohort study, we found that statin use is significantly associated with a decrease in risk of liver disease, HCC, and liver-related deaths. There is limited information on the beneficial associations of statins on liver-healthy individuals, as previous studies^10,14,35^ have focused on chronic liver disease. Our study is the first, to our knowledge, to show a hepatoprotective association of statins in the general population, which may be time, dose, and risk dependent.

One explanation for the potential hepatoprotective association of statins is the inhibition of the prenylation of small guanosine triphosphate hydrolases, which leads to reduced inflammation.^12^ In nonalcoholic steatohepatitis models, fluvastatin has been shown to reduce the activation of hepatic stellate cells by reducing oxidative stress.^11^ In mouse models, statins counteracted angiogenesis and growth of HCC.^36^ Further effects on tumor development are the inhibition of MYC,^37^ nuclear factor–κB cells,^38^ and the protein kinase B signaling pathway,^39,40^ as well as the reduced production of interleukin 6.^38^

We used the UKB to establish an association of statin intake with hepatoprotection. The UKB is limited by lacking information about the length of statin intake, but it is a very well-characterized cohort with deep phenotyping as well as genetic information. This allowed us to establish a unique approach to the interplay between medication and genetics. Our findings suggest that regular statin use provided a particular benefit for *PNPLA3* rs738409 minor allele carriers. However, the rare *TM6SF2* rs58542926 variant, which is more likely to be harmful to the liver,^41^ showed no statistically significant improvement with statins, which might be attributable to insufficient power. Similarly, SE*RPINA1* rs28929474 was associated with chronic liver disease,^31,32^ but no statin-related beneficial association was observed for minor allele carriers. *HSD17B13* rs72613567 and *MTARC1* rs2642438 variant carriers, which have been associated with lower rates of NAFLD, benefitted less from statins as noncarriers.^28,42^ Nevertheless, it would be desirable to perform the analyses in larger cohorts, especially for homozygous minor allele carriers, to exclude that the lack of significance is attributable to small group sizes.

In addition, the UKB allowed us to study a genetic variant in *CYP3A4*, a key enzyme for statin metabolism,^23^ which was associated with decreased expression of *CYP3A4* messenger RNA.^43^ We demonstrated that the statin-related association on liver disease was enhanced in rs35599367 minor allele carriers. These results point toward a dose-dependent association of statins.

To counteract the limitations of the UKB, we added the PMBB and TriNetX cohorts, which had detailed information on statin intake duration. TriNetX provided the opportunity to confirm the results in a large patient cohort of more than 1.5 million individuals and to examine the association of statins with liver disease development in new users. TriNetX also enrolled individuals with more severe disease, which resulted in a higher rate of HCC cases compared with the UKB. Cross-validation of results and numerous sensitivity analyses thus had the potential to minimize misclassification bias, lead time bias, and end-of-follow-up bias. Addition of the PMBB allowed the examination of the association of statin intake duration on liver disease development in a smaller but ethnically diverse cohort.

A potential barrier to preventive treatment might be statin-associated adverse effects. Therefore, we investigated the development of myopathy^44^ but could not find any significant association for statins, which might be caused by individuals discontinuing statin use at the onset of first myopathy symptoms. Our findings confirm previous studies^45,46^ that have reported an increased HR for diabetes in statin users. Remarkably, although the increased incidence of diabetes is a risk factor for chronic liver disease, it did not affect the benefits of statins with respect to the development of new liver disease or liver death in our study.^47,48^

This cohort study now raises the question of whether statin intake should be recommended to individuals at high risk for liver disease. Taking statins was particularly beneficial in men, individuals with diabetes, and individuals with a high FIB-4 index at baseline, which is a routine clinical score to assess a patient’s risk of liver disease.^34^ Still, we need to confirm these associations in randomized clinical trials before recommending statins for liver disease protection.

### Strengths and Limitations

A major strength of this study was the ability to confirm the results using 3 independent cohorts, which all offered longitudinal data. Nevertheless, our study had some limitations. First, the self-reported drug allocation in the UKB data set offered a potential source of error. Second, most individuals in all cohorts have not been formally screened for liver diseases. Therefore, early stages of liver disease could have remained undetected, or, in the absence of a timely diagnosis, an incorrect group allocation could have occurred. Similarly, subgroup analyses focused on individual liver diseases, and genetic analyses involved some groups with small sample sizes; therefore, the results should be considered exploratory and warrant replication in additional cohorts. Moreover, our study is subject to the potential for type I error arising from multiple comparisons in secondary and subgroup analyses. The observational study design inherently presents the possibility of confounding, which we endeavored to mitigate through various sensitivity analyses. To tackle the issue of confounding arising from prevalent statin use, we conducted analyses within the TriNetX cohort, which exclusively comprised new statin users.^49,50,51^ Moreover, the information on alcohol consumption in the UKB was based on a touchscreen interview, which may have limited reliability. Nevertheless, self-reported alcohol consumption in the UKB has been associated with known gene loci for alcohol consumption.^52^ In the TriNetX and PMBB cohorts, we were unable to include any variables related to alcohol consumption.

## Conclusions

In conclusion, this multinational 3-cohort study indicates a significant association between statin intake and liver disease protection, especially in men, individuals with diabetes, and individuals at (genetic) risk of liver disease. Individuals who fall into specific risk categories should undergo a thorough evaluation to determine whether they have an existing indication for taking statins because they are likely to experience significant benefits from this treatment. Finally, these findings call for clinical trials that evaluate drug repurposing for primary and secondary prevention of liver diseases.

## Notes

Supplement 1. **eAppendix.** Supplementary Information **eFigure 1.** Cumulative Incidence of New Liver Diseases and Liver-Associated Deaths in Patients Without Prior Liver Disease in UKB **eFigure 2.** Metabolic Profile of Statin Users Compared to Non-users in Patients Without Prior Liver Disease in UKB **eTable 1.** Numerical Code of the Medication in UKB **eTable 2.** Statin Intake and the Development of Incident Liver Disease, Hepatocellular Carcinoma and Liver-Related Mortality in Patients Without Prior Liver Disease in UKB (Adjusted for Diet, Alcohol Intake and Socioeconomic Status) **eTable 3.** Analysis of the Basic Characteristics of the Cohort From eTable 2 **eTable 4.** Statin Intake and the Development of Incident Liver Disease, Hepatocellular Carcinoma and Liver-Related Mortality in Patients Without Prior Liver Disease in UKB in an Inverse Probability of Treatment Model **eTable 5.** Influence of CYP3A4 Gene Variant and Statin Intake on Liver Health in UKB Gene Carriers Without Prior Liver Disease **eTable 6.** Associations of Statin Use With the Risk of Incident Liver Disease, Hepatocellular Carcinoma, and Liver-Related Mortality in Individuals Without Prior Liver Disease in Different Risk Constellations in UKB **eTable 7.** Overview of the Metabolites of the Volcano Plots
eReferences

Supplement 2.
Data Sharing Statement
