# Supplementary material for: Association of Statin Use With Risk of Liver Disease, Hepatocellular Carcinoma, and Liver-Related Mortality
Source: JAMA Netw Open. 2023 Jun 26;6(6):e2320222. doi: 10.1001/jamanetworkopen.2023.20222 (PMC10293910; doi:10.1001/jamanetworkopen.2023.20222)
Supplement: Supplement 1. — eAppendix. Supplementary Information eFigure 1. Cumulative Incidence of New Liver Diseases and Liver-Associated Deaths in Patients Without Prior Liver Disease in UKB eFigure 2. Metabolic Profile of Statin Users Compared to Non-users in Patients Without Prior Liver Disease in UKB eTable 1. Numerical Code of the Medication in UKB eTable 2. Statin Intake and the Development of Incident Liver Disease, Hepatocellular Carcinoma and Liver-Related Mortality in Patients Without Prior Liver Disease in UKB (Adjusted for Diet, Alcohol Intake and Socioeconomic Status) eTable 3. Analysis of the Basic Characteristics of the Cohort From eTable 2 eTable 4. Statin Intake and the Development of Incident Liver Disease, Hepatocellular Carcinoma and Liver-Related Mortality in Patients Without Prior Liver Disease in UKB in an Inverse Probability of Treatment Model eTable 5. Influence of CYP3A4 Gene Variant and Statin Intake on Liver Health in UKB Gene Carriers Without Prior Liver Disease eTable 6. Associations of Statin Use With the Risk of Incident Liver Disease, Hepatocellular Carcinoma, and Liver-Related Mortality in Individuals Without Prior Liver Disease in Different Risk Constellations in UKB eTable 7. Overview of the Metabolites of the Volcano Plots eReferences [file jamanetwopen-e2320222-s001.pdf]

Supplementary Online Content

Vell MS, Loomba R, Krishnan A, et al. Association of statin use with risk of liver disease, hepatocellular carcinoma, and liver-related mortality. *JAMA Netw Open*. 2023;6(6):e2320222. doi:10.1001/jamanetworkopen.2023.20222

eAppendix. Supplementary Information
eFigure 1. Cumulative Incidence of New Liver Diseases and Liver-Associated Deaths in Patients Without Prior Liver Disease in UKB
eFigure 2. Metabolic Profile of Statin Users Compared to Non-users in Patients Without Prior Liver Disease in UKB
eTable 1. Numerical Code of the Medication in UKB
eTable 2. Statin Intake and the Development of Incident Liver Disease, Hepatocellular Carcinoma and Liver-Related Mortality in Patients Without Prior Liver Disease in UKB (Adjusted for Diet, Alcohol Intake and Socioeconomic Status)
eTable 3. Analysis of the Basic Characteristics of the Cohort From eTable 2
eTable 4. Statin Intake and the Development of Incident Liver Disease, Hepatocellular Carcinoma and Liver-Related Mortality in Patients Without Prior Liver Disease in UKB in an Inverse Probability of Treatment Model
eTable 5. Influence of CYP3A4 Gene Variant and Statin Intake on Liver Health in UKB Gene Carriers Without Prior Liver Disease
eTable 6. Associations of Statin Use With the Risk of Incident Liver Disease, Hepatocellular Carcinoma, and Liver-Related Mortality in Individuals Without Prior Liver Disease in Different Risk Constellations in UKB
eTable 7. Overview of the Metabolites of the Volcano Plots
eReferences

This supplementary material has been provided by the authors to give readers additional information about their work.

© 2023 Vell MS et al. *JAMA Network Open.*

eAppendix. Supplementary Information

Abbreviations

ApoA1	Apolipoprotein A1
ApoB	Apolipoprotein B
B18	Chronic viral hepatitis
B20	Human immunodeficiency virus disease resulting in infectious and parasitic disease
B21	Human immunodeficiency virus disease resulting in malignant neoplasms
B22	Human immunodeficiency virus disease resulting in other specified diseases
B23	Human immunodeficiency virus disease resulting in other conditions
B24	Unspecified human immunodeficiency virus disease
BMI	Body mass index
C	Cholesterol
C22	Malignant neoplasm of liver and intrahepatic bile ducts
C22.0	Hepatocellular carcinoma
CI	Confidence interval
CYP3A4	Cytochrome P450 3A4
E11	Diabetes mellitus type II
E78	Disorder of lipoprotein metabolism and other lipidaemias
EHR	Electronic Health Records
G72.0	Drug-induced myopathy
GlycA	Glycoprotein acetylation
HCC	Hepatocellular carcinoma
HCO	Health care organization
HDL	High density lipoprotein

© 2023 Vell MS et al. *JAMA Network Open.*

HIV	Human immunodeficiency virus
HMG-CoA	3-Hydroxy-3-Methylglutaryl Coenzyme A
HR	Hazard ratio
HSD17B13	Hydroxysteroid 17-Beta Dehydrogenase 13
I10	Essential (primary) hypertension
I11	Hypertensive heart disease
I12	Hypertensive chronic kidney disease
I13	Hypertensive heart and chronic kidney disease
I15	Secondary hypertension
I20	Angina pectoris
I25	Chronic ischaemic heart disease
ICD-10	International Classification of Diseases and Related Health Problems
IDL	Intermediate density lipoprotein
K70	Alcohol-associated liver disease
K71	Toxic liver disease
K72	Hepatic failure, not elsewhere classified
K73	Chronic hepatitis, not elsewhere classified
K74	Fibrosis and cirrhosis of liver
K75	Other inflammatory liver diseases
K76	Other diseases of liver
K77	Liver disorders in diseases classified elsewhere
LDL	Low density lipoprotein
LMWH	Low molecular weight heparin
MTARC1	Mitochondrial Amidoxime Reducing Component1
MUFA	Monounsaturated fatty acids

© 2023 Vell MS et al. *JAMA Network Open.*

NAFLD	Non-alcoholic fatty liver disease
NASH	Non-alcoholic steatohepatitis
OR	Odds ratio
PL	Phospholipids
PMBB	Penn Medicine Biobank
PNPLA3	Patatin-like phospholipase domain-containing protein 3
PS	Propensity score
PUFA	Polyunsaturated fatty acids
SD	Standard deviation
SERPINA1	Serpin family A member 1
SMD	Standardized mean difference
SNP	Single nucleotide polymorphism
TG	Triglycerides
TriNetX	TriNetX Network
TNX	TriNetX
TM6SF2	Transmembrane 6 superfamily member 2
UK	United Kingdom
UKB	UK Biobank
VLDL	Very low density lipoprotein
Z94.4	Liver transplant status

© 2023 Vell MS et al. *JAMA Network Open.*

UK Biobank

Enrollment

The UKB included patients aged 37-73 years who were recruited from 2006-2010 until May 2021 (end of follow-up). Written consent for data linkage and genotyping was obtained from all participants. Each participant was enrolled by the UK National Health Service and was initially examined as part of the enrollment process. Regular long-term examinations were also conducted.

Exclusion criteria

Criteria for exclusion from the UK Biobank cohort were missing BMI data, HIV (B20-B24) or chronic viral hepatitis (B18), which affected 3932 patients (Figure 1).

We excluded 19 patients with HCC, 2125 patients with liver disease and 4396 patients with pathological alcohol consumption at baseline (Figure 1). One patient was excluded due to a lack of survival data (Figure 1).

Medication

Health professionals conducted interviews to register medications, which were then numerically coded. Of the 4382 medications recorded, 4199 were included.

Patients' medication codes data was sorted and scored according to medication type (i.e., statin, beta-blocker, proton-pump-inhibitor, etc). Unclearly titled specific names and duplicate alignments of specific names were excluded from the study.

Metabolic profile of statin-users compared to non-users in patients without prior liver disease in UKB

Statins are primarily used for their lipid-lowering properties, which protect against serious cardiovascular events and strokes.[1] Würtz et al. analyzed the metabolic profile of patients taking statins. It was shown that the initiation of statin use led to an 80%

© 2023 Vell MS et al. *JAMA Network Open.*

reduction in remnant cholesterol relative to LDL cholesterol. Among fatty acids, omega-6 fatty acids decreased the most.[2] We were able to confirm these results in our study (**Table S7**).

Ethnicity

Ethnicity was also considered in matching and numerical values were used to assign patients. Black (‘Caribbean’, African’, ‘Any other black backgorund’), Asian(‘Indian’, ‘Pakistani’, ‘Bangladeshi’, ‘Any other asian background’), White (‘British’, ‘Irish’, ‘Any other white background’) and other ethnicities (‘Mixed’, ‘Chinese’, ‘Other ethnic group’) were represented.[3]

Penn Medicine Biobank

Enrollment

The PMBB included patients aged 18-102 years with ongoing recruitment. End of follow-up was December 2020. The PMBB consists of patients enrolled from phlebotomy sites throughout the health system and is agnostic to their underlying illness.

Exclusion criteria

Criteria for exclusion from the PMBB cohort were incorrect (BMI<5 or >500 kg/m^2) or missing BMI data and incorrect (negative or <18 years) or missing age and survival data (n=24,337). In addition, we excluded a further 373 patients due to HIV infection (B20). No HIV-associated diseases (B21-B24) were reported. Finally, we excluded 1214 cases due to chronic hepatitis (B18) (Figure 1).

Medication

-

© 2023 Vell MS et al. *JAMA Network Open.*

Ethnicity

Ethnicity in PMBB was also considered in matching, and numerical values were used to match patients. In PMBB, Black, Asian, American Indian and Alaska native, White and other ethnicities (Hispanic and Pacific Island, Other or Unknown) were represented.

TriNetX

Enrollment

The TriNetX research network (Cambridge, MA) provides access to the EHRs of patients from health care organizations (HCOs) in the United States. Patients in TriNetX were 18-90 years old and were enrolled with real-time data between January 2011 and December 2020. End of follow-up was September 2022. All clinical variables are retrieved directly from the EHR through an integrated system of clinical records. TriNetX can also extract facts of interest from the narrative text of clinical documents using natural language processing. Data are mapped to a standard and controlled set of clinical terminologies and converted to a proprietary data schema. This transformation process includes an extensive data quality assessment to reject records that do not meet quality standards. Quality assurance of the data is performed using a standardized format before integration into the database. Patient data is reduced to statistical summaries so that patients remain de-identified at all times. Likewise, the source of the individual healthcare system remains unknown. Death register was used to obtain information on mortality and the respective age of the individual.

Statistical analysis

All statistical analyses were performed in real-time using the TriNetX platform. We first compared baseline patient characteristics. Categorical variables were compared using chi-squared tests, and continuous variables were assessed using an independent-

© 2023 Vell MS et al. *JAMA Network Open.*

samples t-test. Analyses were performed to examine the risk of study outcomes using Cox proportional hazards models. HR and CI along with tests for proportionality were calculated using R's Survival package v3.2-3 and numbers were then validated by comparing them with the output from SAS version 9.4. An a priori-defined 2-sided alpha of <0.05 was used for statistical significance.

a) Development of the Propensity Score Model

Each patient taking statins regularly was matched to a patient in the control group using 1:1 propensity score matching to reduce confounding effects. For the liver-healthy cohort, the propensity score model was adjusted for the following variables: age, sex, BMI, E11, I10, E78.

Logistic regression on these input matrices was used to obtain propensity scores for each patient in both cohorts. Logistic regression was performed in Python 3.6.5 (Python Software Foundation) using standard libraries NumPy and sklearn. The same analyses were also performed in R 3.4.4 software (R Foundation for Statistical Computing, Vienna, Austria) to ensure outputs match. After calculating propensity scores, matching was performed using nearest-neighbor matching algorithm with a caliper of 0.1 pooled standard deviations. The order of the rows in the covariate matrix can affect the nearest neighbor matching; therefore, the order of the rows in the matrix was randomized to eliminate this bias.

© 2023 Vell MS et al. *JAMA Network Open.*

eFigure 1. Cumulative Incidence of New Liver Diseases and Liver-Associated Deaths in Patients Without Prior Liver Disease in UKB

A

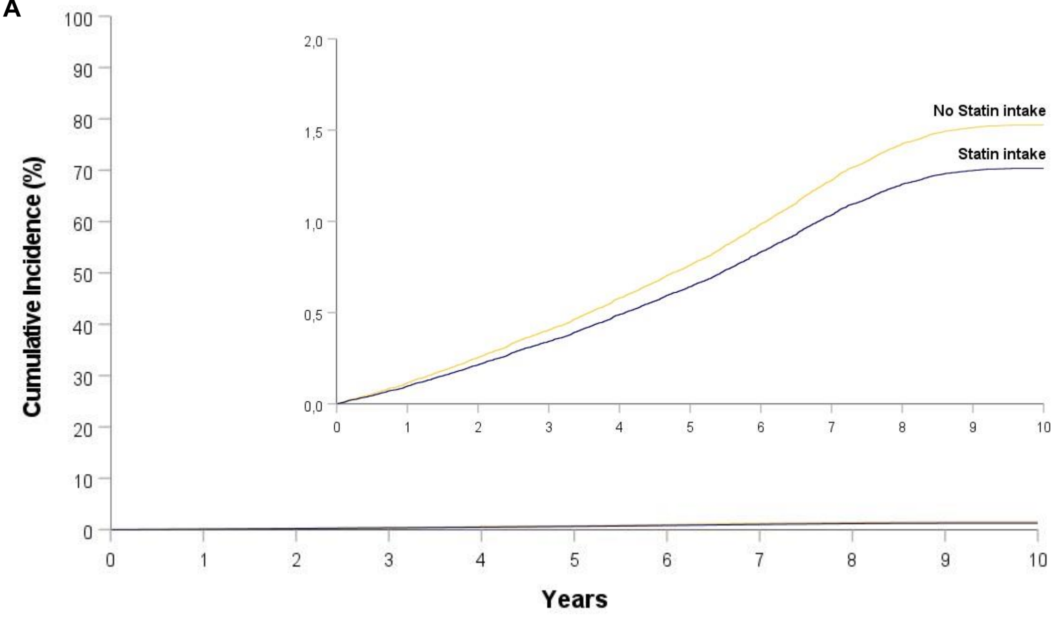

No. at Risk						
No Statin intake	148,948	147,950	146,424	144,446	142,022	139,078
Statin intake	56,109	55,724	55,100	54,350	53,376	52,180

A. Cumulative incidence of new liver diseases (K70-K77) in patients without prior liver disease in UKB. Alternative event was death or end of follow-up. To depict the cumulative incidence of new liver disease under Statin intake, we used the Cox Regression model. We adjusted for the variables age, sex, BMI, ethnicity, number of medications and Statin use.

B

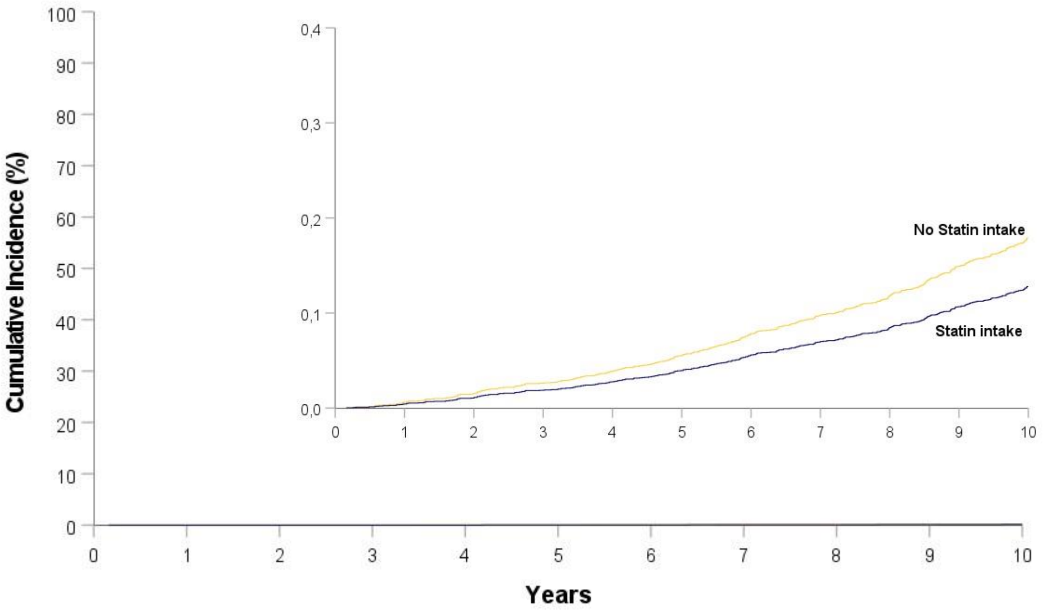

No. at Risk						
No Statin intake	148,948	147,950	146,424	144,446	142,022	139,078
Statin intake	56,109	55,724	55,100	54,350	53,376	52,180

B. Cumulative incidence of liver-associated deaths in patients without prior liver disease in UKB. Alternative event was death from non-liver-related causes or end of follow-up. To depict the cumulative incidence of liver-related deaths under Statin intake, we used the Cox Regression model. We adjusted for the variables age, sex, BMI, ethnicity, number of medications and Statin use.

© 2023 Vell MS et al. *JAMA Network Open.*

eFigure 2. Metabolic Profile of Statin Users Compared to Non-users in Patients Without Prior Liver Disease in UKB

Lipoprotein subclasses particle concentrations

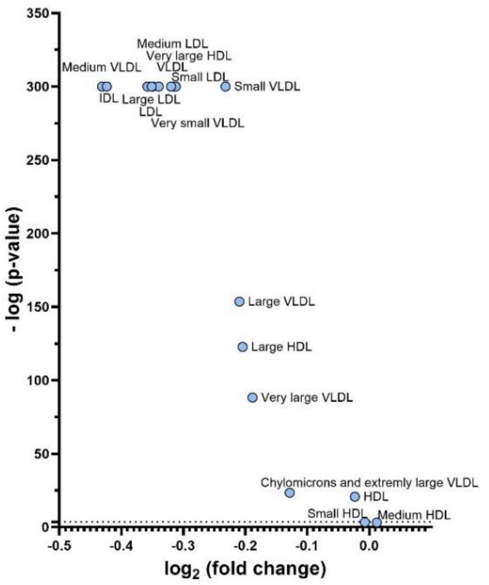

Cholesterol, Triglycerides and Apolipoproteins

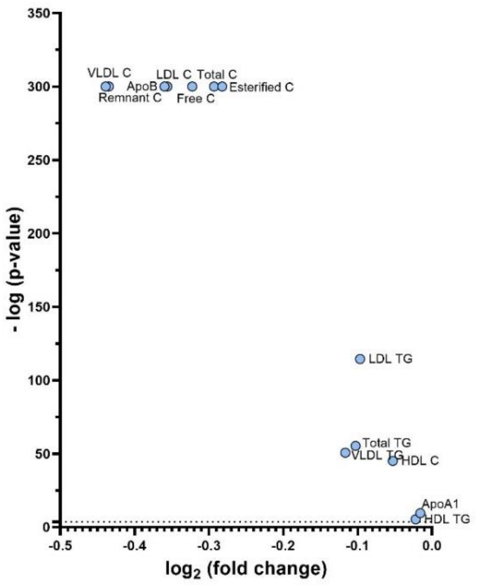

Fatty acids

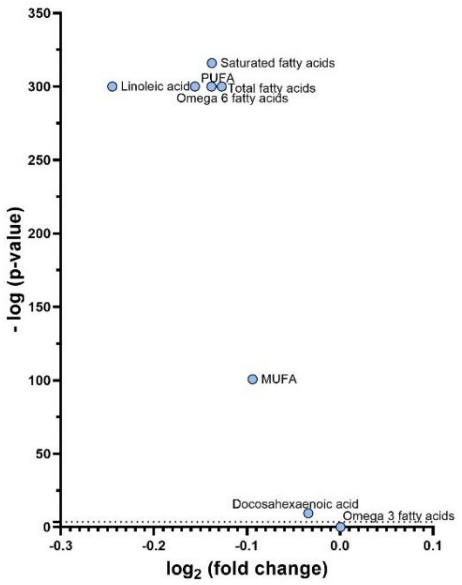

Phospholipids and miscellaneous

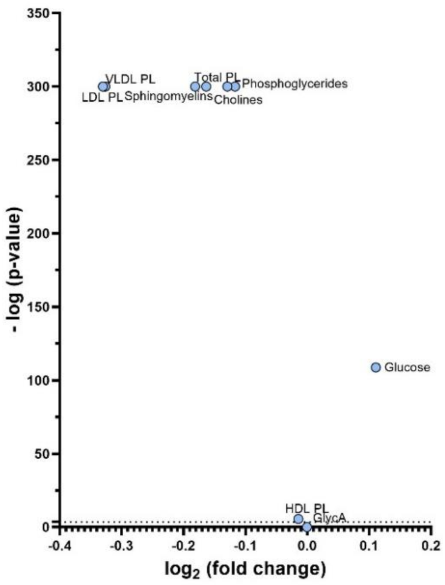

Figure S2. Metabolic profile of statin-users compared to non-users in patients without prior liver disease in UKB - Abbreviations: ApoA1, Apolipoprotein A1; ApoB, Apolipoprotein B'; C, Cholesterol; GlycA, Glycoprotein acetylation; HDL, High-density lipoprotein; IDL, Intermediate density lipoprotein; LDL, Low-density lipoprotein; MUFA, Monounsaturated fatty acids; PL, Phospholipids; PUFA, Polyunsaturated fatty acids; TG, Triglycerides; VLDL, Very-low-density lipoprotein.

© 2023 Vell MS et al. *JAMA Network Open.*

**eTable 1.** Numerical Code of the Medication in UKB		
Medication group	Subgroup	Numerical Code
Aspirin[a]		1140909772, 1140861804, 1140868226, 1140861806, 1140882392, 1140882268, 1140882108, 1140882190, 1140868282, 1140872040, 1141163138, 1141167848, 1140909888, 1140871080, 1140925942, 1140923344, 1140856336, 1141167844, 1140861808, 1140882192, 1141151924, 1140868264, 1140882106, 1140856394, 1141164050, 1141164044, 1140856314, 1140863514, 1140856220, 1141177826, 1140872032, 1140864860, 1140856212, 1141188536, 1140917408, 1140868294, 1140856440, 1140856214, 1140856344
Biguanide [a]	Metformin	1140921964, 1140874686, 1140884600, 1141189090, 1141153138
Insulin		1140883066
Statin	Atorvastatin, Fluvastatin, Pravastatin, Rosuvastatin, Simvastatin	1141146234, 1141192414, 1140910632, 1140888594, 1140864592, 1141146138, 1140861970, 1140888648, 1141195196, 1141192410, 1141188146, 1140861958, 1140910652, 1140910654, 1140881748, 1141200040

[a] Combined preparations.

© 2023 Vell MS et al. *JAMA Network Open.*

**eTable 2.** Statin Intake and the Development of Incident Liver Disease, Hepatocellular Carcinoma and Liver-Related Mortality in Patients Without Prior Liver Disease in UKB (Adjusted for Diet, Alcohol Intake and Socioeconomic Status)[A]			
Event and Treatment Group	No. with Event/ Total No.	Hazard Ratio (95% CI)	P-value
New Liver Disease (K70-K77)			
No Statin intake	1995/131,128	1.00 (reference)	-
Statin intake	768/49,309	0.84 (0.77 to 0.91)	<.001[c]
Subdiagnoses[b]			
Alcohol-associated liver disease (K70)	87/49,309	0.82 (0.63 to 1.07)	.1
Toxic liver disease (K71)	3/49,309	0.28 (0.08 to 0.93)	.04[c]
Hepatic failure, not elsewhere classified (K72)	62/49,309	0.88 (0.65 to 1.20)	.4
Chronic hepatitis, not elsewhere classified (K73)	11/49,309	1.28 (0.63 to 2.61)	.5
Fibrosis and cirrhosis of liver (K74)	106/49,309	0.73 (0.58 to 0.91)	.006[c]
Other inflammatory liver diseases (K75)	92/49,309	0.80 (0.63 to 1.02)	.07
Other diseases of liver (K76)	597/49,309	0.87 (0.79 to 0.96)	.005[c]
Liver disorders in diseases classified elsewhere (K77)	1/49,309	0.62 (0.05 to 8.02)	.7
Liver cell carcinoma (C220)	21/49,309	0.68 (0.41 to 1.12)	.1
Liver transplant status (Z944)	7/49,309	0.63 (0.27 to 1.50)	.3
Liver-related Death			
No Statin intake	344/131,128	1.00 (reference)	-
Statin intake	129/49,309	0.77 (0.62 to 0.95)	.01[c]

[a] Competing risk analysis was performed by additional correction for socioeconomic status (Townsend index), alcohol consume in g/d, and diet (Vegetables and Fruit intake per day, Fish intake per week and Meat intake per week). For this purpose, 57,377 patients were excluded before matching due to missing data.

[b] For subdiagnoses, only patients taking statins were referred to, with hazard ratios and P-values calculated consistently compared to patients not taking statins.

[c] Significant P-value.

© 2023 Vell MS et al. *JAMA Network Open.*

**eTable 3.** Analysis of the Basic Characteristics of the Cohort From eTable 2				
Patients without prior liver disease in UKB (adapted for diet, alcohol intake and socioeconomic status)	No Statin intake (N=131,128)	Statin intake (N=49,309)	Standardized mean difference before PS	Standardized mean difference after PS
Age (Years)	60±6.5	61±6.2	0.8	0.1
Sex (% Women)	48	45	0.4	0.0
BMI (kg/m^2)	28.2±5.0	28.8±4.7	0.5	0.0
Ethnicity (% White)	96	95	0.0	0.0
Number of medications	3.8±3.0	5.0±3.0	1.3	0.0
Diabetes mellitus type II (E11)	6	13	0.6	0.0
Arterial hypertension (I10)	31	44	0.9	0.0
Disorders of lipoprotein metabolism and other lipidaemias (E78)	13	27	0.9	0.0
			P-value before PS	P-value after PS
Alcohol consumption (g/d)	8.96±10.37	9.26±10.75	<.001[a,b]	<.001[a,b]
Vegetables and Fruit intake per day (tablespoon/pieces)	7.5±4.1	7.5±4.0	<.001[a,b]	.8[a]
Fish intake per week	3.6±1.4	3.6±1.4	<.001[a,b]	<.001[a,b]
Meat intake per week	8.0±2.7	8.1±2.6	<.001[a,b]	<.001[a,b]
Socioeconomic Status (Townsend Index)	-1.44±3.03	-1.21±3.14	<.001[a,b]	<.001[a,b]

[a] Univariate P-values were obtained for continuous variables using an independent T-test.

[b] Significant P-value.

© 2023 Vell MS et al. *JAMA Network Open.*

eTable 4. Statin Intake and the Development of Incident Liver Disease, Hepatocellular Carcinoma and Liver-Related Mortality in Patients Without Prior Liver Disease in UKB in an Inverse Probability of Treatment Model

Event and Treatment Group	No. with Event/ Total No.	Hazard Ratio (95% CI)	P-value
New Liver Disease[a]			
No Statin intake	3968/411,377	1.00 (reference)	-
Statin intake	1605/80,661	0.69 (0.48 to 0.97)	.03[c]
Subdiagnoses[b]			
Alcohol-associated liver disease (K70)	168/80,661	0.72 (0.50 to 1.04)	.08
Toxic liver disease (K71)	13/80,661	0.46 (0.20 to 1.03)	.06
Hepatic failure, not elsewhere classified (K72)	139/80,661	0.85 (0.57 to 1.27)	.4
Chronic hepatitis, not elsewhere classified (K73)	18/80,661	0.79 (0.42 to 1.50)	.5
Fibrosis and cirrhosis of liver (K74)	243/80,661	0.76 (0.59 to 0.98)	.03[c]
Other inflammatory liver diseases (K75)	201/80,661	1.31 (0.81 to 2.12)	.3
Other diseases of liver (K76)	1236/80,661	1.10 (0.90 to 1.34)	.3
Liver disorders in diseases classified elsewhere (K77)	4/80,661	0.45 (0.10 to 2.10)	.3
Liver cell carcinoma (C22.0)	45/80,661	0.55 (0.35 to 0.87)	.01[c]

[a] Incident Liver Disease is defined as new onset Liver Disease K70-K77 or C22.0 after Baseline examination.
[b] For subdiagnoses, only patients taking statins were referred to, with hazard ratios and P-values calculated consistently compared to patients not taking statins.
[c] Significant P-value.

© 2023 Vell MS et al. *JAMA Network Open.*

eTable 5. Influence of *CYP3A4* Gene Variant and Statin Intake on Liver Health in UKB Gene Carriers Without Prior Liver Disease

Event and Treatment Group	No. with Event/ Total No.	Hazard Ratio (95% CI)	P-value
rs35599367			
New Liver Disease (K70-K77)	75/5354	0.68 (0.51 to 0.89)	.005[a]
Liver-related Death	11/5354	0.55 (0.27 to 1.13)	.1
Incident HCC	0/5354	-	-

Heterozygous and homozygous gene carriers were considered. Separate matching was performed at a 2:1 ratio.
[a] Significant P-value.

© 2023 Vell MS et al. *JAMA Network Open.*

eTable 6. Associations of Statin Use With the Risk of Incident Liver Disease, Hepatocellular Carcinoma, and Liver-Related Mortality in Individuals Without Prior Liver Disease in Different Risk Constellations in UKB[a]

	No. Event/ Total No.*	Hazard Ratio (95% CI)	P-value	No. Event/ Total No.*	Hazard Ratio (95% CI)	P-value	No. Event/ Total No.*	Hazard Ratio (95% CI)	P-value
	Liver Disease (K70-K77)			Incident HCC			Liver-related Death		
in Men[b]	502/31,542	0.76 (0.68 to 0.85)	<.001[d]	16/31,542	0.53 (0.30 to 0.95)	.03[d]	87/31,542	0.63 (0.49 to 0.82)	<.001[d]
In Women[b]	388/24,567	0.94 (0.83 to 1.05)	.3	5/24,567	0.68 (0.25 to 1.86)	.5	51/24,567	0.89 (0.65 to 1.24)	.5
FIB-4 <1.3[c]	293/21,728	0.85 (0.75 to 0.98)	.02[d]	1/21,728	0.17 (0.02 to 1.35)	.09	35/21,728	0.85 (0.57 to 1.27)	.4
FIB-4 1.3-2.67[c]	426/27,686	0.89 (0.79 to 0.996)	.04[d]	13/27,686	0.76 (0.38 to 1.49)	.4	61/27,686	0.67 (0.49 to 0.91)	.009[d]
FIB-4 >2.67[c]	90/1749	0.70 (0.55 to 0.90)	.006[d]	6/1749	0.57 (0.22 to 1.51)	.3	31/1749	0.71 (0.47 to 1.08)	.1
Diabetes mellitus type II (E11)	324/7590	0.66 (0.57 to 0.76)	<.001[d]	13/7590	0.56 (0.27 to 1.14)	.11	50/7590	0.61 (0.42 to 0.89)	.01[d]
PNPLA3 rs738409 (wt)	494/33,792	0.88 (0.79 to 0.98)	.02[d]	12/33,792	0.78 (0.40 to 1.51)	.5	69/33,792	0.66 (0.50 to 0.87)	.004[d]
PNPLA3 rs738409 (het)	310/18,430	0.82 (0.72 to 0.94)	.004[d]	5/18,430	0.31 (0.11 to 0.85)	.02[d]	50/18,430	0.77 (0.55 to 1.07)	.1
PNPLA3 rs738409 (hom)	56/2448	0.73 (0.53 to 1.00)	.05	4/2448	0.87 (0.24 to 3.11)	.8	15/2448	0.89 (0.46 to 1.72)	.7
TM6SF2 rs58542926 (wt)	723/47,423	0.85 (0.78 to 0.93)	<.001[d]	16/47,423	0.65 (0.36 to 1.18)	.2	109/47,423	0.70 (0.56 to 0.88)	.002[d]
TM6SF2 rs58542926 (het)	129/6955	0.86 (0.70 to 1.06)	.2	5/6955	0.52 (0.20 to 1.37)	.2	19/6955	0.61 (0.37 to 1.01)	.06
TM6SF2 rs58542926 (hom)	5/191	0.78 (0.28 to 2.15)	.6	0/191	-	-	3/191	6.48 (0.98 to 43.00)	.05
HSD17B13 rs72613567 (wt)	452/29,083	0.80 (0.71 to 0.89)	<.001[d]	13/29,083	0.61 (0.31 to 1.20)	.2	79/29,083	0.73 (0.55 to 0.95)	.02[d]
HSD17B13 rs72613567 (het)	333/21,352	0.88 (0.77 to 1.00)	.06	7/21,352	0.63 (0.27 to 1.47)	.3	46/21,352	0.69 (0.49 to 0.98)	.04[d]
HSD17B13 rs72613567 (hom)	73/4069	1.06 (0.79 to 1.43)	.7	1/4069	0.25 (0.03 to 2.07)	.2	8/4069	0.62 (0.27 to 1.43)	.3

© 2023 Vell MS et al. *JAMA Network Open.*

MTARC1 rs2642438 (wt)	446/27,751	0.85 (0.76 to 0.95)	.005[d]	13/27,751	0.67 (0.34 to 1.29)	.2	83/27,751	0.79 (0.61 to 1.03)	.08
MTARC1 rs2642438 (het)	345/22,330	0.84 (0.74 to 0.96)	.008[d]	7/22,330	0.54 (0.23 to 1.27)	.2	45/22,330	0.61 (0.43 to 0.8)	.005[d]
MTARC1 rs2642438 (hom)	69/4545	0.85 (0.63 to 1.13)	.3	1/4545	0.25 (0.03 to 1.96)	.2	6/4545	0.62 (0.24 to 1.60)	.3
SERPINA1 rs28929474 (wt)	854/54,140	0.85 (0.79 to 0.92)	<.001[d]	19/54,140	0.61 (0.36 to 1.03)	.1	127/54,140	0.72 (0.58 to 0.89)	.002[d]
SERPINA1 rs28929474 (het)	36/1962	0.78 (0.52 to 1.19)	.3	2/1962	0.50 (0.06 to 4.19)	.5	11/1962	0.85 (0.39 to 1.83)	.7
SERPINA1 rs28929474 (hom)	0/7	-	-	0/7	-	-	0/7	-	-

[a] For sensitivity analyses, only individuals taking statins were reported, with hazard ratios and P-values calculated consistently compared to individuals not taking statins.

[b] Sex was excluded from the covariates.

[c] Classification by 'Development of a simple noninvasive index to predict significant fibrosis in individuals with HIV/HCV coinfection'. [34]

[d] Significant P-value

© 2023 Vell MS et al. *JAMA Network Open.*

**eTable 7.** Overview of the Metabolites of the Volcano Plots		
Metabolic profile	Log_2 (fold change)	Log (P-value)
Saturated fatty acids	-.138	316.03
Total C	-.293	300.00
Remnant C	-.435	300.00
VLDL C	-.439	300.00
LDL C	-.356	300.00
Total PL	-.163	300.00
VLDL PL	-.326	300.00
LDL PL	-.330	300.00
Esterified C	-.282	300.00
Free C	-.323	300.00
VLDL	-.314	300.00
LDL	-.350	300.00
Phosphoglycerides	-.117	300.00
Cholines	-.129	300.00
Sphingomyelins	-.181	300.00
ApoB	-.360	300.00
Total fatty acids	-.127	300.00
Omega 6 fatty acids	-.156	300.00
PUFA	-.138	300.00
Linoleic acid	-.245	300.00
Medium VLDL	-.431	300.00
Small VLDL	-.232	300.00
Very small VLDL	-.340	300.00
IDL	-.424	300.00
Large LDL	-.358	300.00
Medium LDL	-.351	300.00
Small LDL	-.312	300.00
Very large HDL	-.320	300.00
Large VLDL	-.209	153.57
Large HDL	-.204	122.70
LDL TG	-.097	114.45
Glucose	.111	108.70
MUFA	-.094	100.68
Very large VLDL	-.188	88.20
Total TG	-.103	55.27
Alanine	.059	55.09
VLDL TG	-.117	50.65
Albumin	.019	47.04
HDL C	-.053	45.16
Leucine	.052	36.90
Branched-chain amino acids	.039	27.94
Chylomicrons and extremly large VLDL	-.128	23.41
Valine	.031	23.34

© 2023 Vell MS et al. *JAMA Network Open.*

HDL	-.023	20.72
Glycine	-.051	17.60
Isoleucine	.045	17.42
Creatinine	.032	16.48
Degree of unsaturation	.006	9.98
ApoA1	-.016	9.47
Docosahexaenoic acid	-.034	9.44
Lactase	.026	8.97
Tyrosine	.020	8.52
Histidine	-.013	6.74
HDL PL	-.014	5.64
HDL TG	-.022	5.27
Phenylalanine	.016	4.84
Small HDL	-.007	3.39
Medium HDL	.012	3.16
Pyruvate	.017	2.37
Glutamine	-.004	1.31
Acetate	-.048	.95
Citrate	-.004	.73
GlycA	.000	.08
Omega 3 fatty acids	.001	.04

Abbreviations: ApoA1, Apolipoprotein A1; ApoB, Apolipoprotein B; C, Cholesterol; GlycA, Glycoprotein acetylation; HDL, High-density lipoprotein; IDL, Intermediate density lipoprotein; LDL, Low-density lipoprotein; MUFA, Monounsaturated fatty acids; PL, Phospholipids; PUFA, Polyunsaturated fatty acids; TG, Triglycerides; VLDL, Very-low-density lipoprotein.

© 2023 Vell MS et al. *JAMA Network Open.*

eReferences

1. Efficacy and safety of cholesterol-lowering treatment: Prospective meta-analysis of data from 90 056 participants in 14 randomised trials of statins. *Lancet*. 2005;366(9493). doi:10.1016/S0140-6736(05)67394-1

2. Würtz P, Wang Q, Soininen P, et al. Metabolomic Profiling of Statin Use and Genetic Inhibition of HMG-CoA Reductase. *J Am Coll Cardiol*. 2016;67(10). doi:10.1016/j.jacc.2015.12.060

3. UK Biobank. Published January 20, 2022. Accessed January 25, 2022. https://www.ukbiobank.ac.uk/

© 2023 Vell MS et al. *JAMA Network Open.*
