# Supplementary material for: Association of Statin Use With Risk of Liver Disease, Hepatocellular Carcinoma, and Liver-Related Mortality
Source: JAMA Netw Open. 2023 Jun 26;6(6):e2320222. doi: 10.1001/jamanetworkopen.2023.20222 (PMC10293910; doi:10.1001/jamanetworkopen.2023.20222)
Supplement: Supplement 2. — Data Sharing Statement [file jamanetwopen-e2320222-s002.pdf]

Data Sharing Statement

Vell. Association of Statin Use With Risk of Liver Disease, Hepatocellular Carcinoma, and Liver-Related Mortality. *JAMA Netw Open*. Published June 26, 2023.
doi:10.1001/jamanetworkopen.2023.20222

Data
Data available: No
